# Fast EM-driven nature-inspired optimization of antenna input characteristics using response features and variable-resolution simulation models

**DOI:** 10.1038/s41598-024-60749-5

**Published:** 2024-05-02

**Authors:** Slawomir Koziel, Anna Pietrenko-Dabrowska

**Affiliations:** 1 Engineering Optimization and Modeling Center, Reykjavik University, 102 Reykjavik, Iceland; 2 Faculty of Electronics, Telecommunications and Informatics, Gdansk University of Technology, 80-233 Gdansk, Poland

**Keywords:** Antenna design, Global optimization, Surrogate modeling, Response features, Kriging, Nature-inspired algorithms, Multi-resolution EM analysis, Electrical and electronic engineering, Computational science

## Abstract

Utilization of optimization technique is a must in the design of contemporary antenna systems. Often, global search methods are necessary, which are associated with high computational costs when conducted at the level of full-wave electromagnetic (EM) models. In this study, we introduce an innovative method for globally optimizing reflection responses of multi-band antennas. Our approach uses surrogates constructed based on response features, smoothing the objective function landscape processed by the algorithm. We begin with initial parameter space screening and surrogate model construction using coarse-discretization EM analysis. Subsequently, the surrogate evolves iteratively into a co-kriging model, refining itself using accumulated high-fidelity EM simulation results, with the infill criterion focusing on minimizing the predicted objective function. Employing a particle swarm optimizer (PSO) as the underlying search routine, extensive verification case studies showcase the efficiency and superiority of our procedure over benchmarks. The average optimization cost translates to just around ninety high-fidelity EM antenna analyses, showcasing excellent solution repeatability. Leveraging variable-resolution simulations achieves up to a seventy percent speedup compared to the single-fidelity algorithm.

## Introduction

Development of antennas is a difficult undertaking for a number of reasons. On the one hand, existing and emerging application areas^1–5^ dictate strict performance requirements, related to both electrical and field parameters (broadband, and multi-band operation, high gain, circular polarization, beam scanning, or MIMO operation^6–11^), reconfigurability^12^, but also small physical dimensions^13–16^. On the other hand, handling of complex antenna geometries developed to fulfil these needs^17–20^ is a considerable challenge by itself. For example, parametric studies, still widely employed for dimension adjustment, are insufficient to control multiple variables, let alone several design objectives or constraints. Instead, rigorous numerical optimization methods are recommended^21,22^. However, accurate evaluation of antenna characteristics demands electromagnetic (EM) analysis, known for its computational cost. EM-driven optimization often demands numerous antenna evaluations, leading to potentially prohibitive computational expenses. Even local parameter tuning, whether using gradient-^23^ or stencil-based techniques^24^, may necessitate dozens or even hundreds of antenna simulations. Global optimization^25^, as well as other procedures (multi-criterial design^26^, statistical analysis^27^, tolerance optimization^28^), are incomparably more expensive when executed directly using EM simulation models.

Despite the mentioned challenges, global search is more and more often recommended if not necessary. This is the case for inherently multimodal tasks (array pattern optimization^29^, frequency-selective surface development^30^, design of metamaterials^31^), design of compact antennas based on topological modifications (stubs^32^, defected ground structures^33^, shorting pins^34^) leading to parameter redundancy and parameter space enlargement^35^, antenna redesign over wide operating parameters ranges^36^, or simply unavailability of a good starting point. In modern times, global optimization primarily revolves around nature-inspired metaheuristic algorithms^37–39^. These algorithms operate on sets (populations)^40^ of potential solutions (individuals, agents)^41^ rather than single parameter vectors. Their global search capability stems from exchanging information among the population members, either by communicating the most promising parameter space locations^42^, sharing information within individuals^43^, or integrating stochastic components. The latter might be in a form of partially random selection procedures^44^, but also random modifications of the parameter vectors^45^. There is a large variety of nature-inspired procedures available. Widely used methods encompass genetic/evolutionary algorithms, firefly algorithm, differential evolution, particle swarm optimizers (PSO), ant systems, grey wolf optimization^46–53^, and a plethora of others^54–57^. The algorithms of this class have a simple structure and are straightforward to handle. However, in terms of EM-based design they are impractical due to poor computational efficiency. An average optimization run entails several thousand evaluations of the objective function, which can be restrictive, especially for moderately or highly complex antenna structures. As a result, direct application of nature-inspired optimization methods becomes feasible only when EM analysis is relatively rapid or when hardware and licensing enable parallel processing.

Nowadays, one of the most popular ways of enabling nature-inspired optimization of expensive simulation models are surrogate modeling techniques^58–60^. A standard surrogate-assisted framework operates iteratively, relying on a surrogate model as a predictor to approximate the optimal design^61^. Subsequently, the simulation data gathered during the process aids in refining this surrogate. New data points, often termed infill points, are generated based on diverse criteria aiming to enhance model accuracy across the parameter space (exploration^62^), pinpoint the optimal design (exploitation^63^), or enable balanced exploration and exploitation^64^). The nature-inspired algorithm is tasked with optimizing either the surrogate model or the predicted modeling error^65^. The specific modeling methods often used for this purpose are kriging^66^, Gaussial Process Regression (GPR)^67^, or neural networks^68^. Recently, the frameworks of this type have been often referred to as machine learning (ML) procedures^69,70^. A comprehensive review of ML techniques for antenna design can be found in^99^. The performance of surrogate-assisted nature-inspired algorithms is hindered by problems related to a rendition of an accurate metamodel, which is particularly troublesome for highly-nonlinear antenna responses. The challenge becomes more pronounced when aiming for a model that remains valid across broad ranges of design variables and frequencies. Available algorithms often demonstrate their effectiveness using low-dimensional examples (typically up to six parameters)^71,72^. Available mitigation approaches include performance-driven modeling^36,73,74^, although its incorporation into global optimization procedures is not straightforward. Other possibilities are variable-resolution EM simulations (e.g., initial pre-screening executed using the low-fidelity analysis^75^, or utilization of co-kriging models^76^), and response feature technology^77^ (mainly employed for local tuning^78^, but also generic surrogate modeling frameworks^79^). The approach involves reframing the problem at hand using system output characteristics (features), such as frequency or level coordinates of resonances. These coordinates have a less nonlinear dependence on design variables compared to the entire frequency characteristics. This strategy offers a significant simplification of the design task^80^, leading to faster convergence^81^ or a decrease in the number of training samples necessary to identify a dependable metamodel^82^.

This work introduces an innovative surrogate-based technique for cost-effective global design enhancement of multi-band antenna devices. The presented approach leverages response feature technology and variable-resolution EM simulations. Initially, a kriging metamodel is constructed based on response features extracted from a set of random observables, allowing determination of attractive regions of the parameter space at low cost through low-fidelity EM analysis. Subsequent surrogate refinement relies on infill points generated by optimizing the metamodel. The optimization is executed using a particle swarm optimizer, minimizing the predicted objective function. Maintaining accuracy entails high-fidelity EM analysis at this stage, constructing the surrogate model through co-kriging. Using response features further reduces the optimization's computational cost. Extensive verification experiments demonstrate the relevance and effects of our framework's algorithmic mechanisms on both reliability and computational efficiency. Comparisons with direct nature-inspired optimization, surrogate-assisted algorithms working with complete antenna responses, and single-resolution feature-based methods confirm significant reduction in runtime without compromising design quality. Depending on the test case, the overall optimization cost corresponds to as few as 120 high-fidelity EM simulations, showing excellent solution repeatability.

## Global antenna optimization by response features and variable-resolution simulation models

Here, we present our optimization technique. Section "Design problem formulation" revisits the formulation of the design task, specifically focusing on optimizing multi-band antenna input characteristics. The concept of response features is outlined in Section "Response features". Section "Variable-resolution EM models" delves into variable-resolution EM models, while Section "Kriging interpolation. Co-Kriging Surrogates" formulates kriging and co-kriging modeling, intended for constructing the initial surrogate (low-fidelity) in Section "Parameter space pre-screening. construction of the initial surrogate" and the refined surrogate (high-fidelity) in Section "Generating infill points using PSO. Co-Kriging surrogate". This refined surrogate is involved in generating infill points through the improvement of the predicted merit function, as detailed in Section "Generating infill points using PSO. Co-Kriging surrogate". The optimization engine used in this stage is a particle swarm optimizer. Finally, Section "Optimization framework" encompasses an extensive summary of the complete design procedure.

At this point, it should be reiterated that the proposed optimization methodology relies on surrogate modeling techniques. The main underlying idea is to replace massive evaluations of antenna characteristics using EM simulations with their predictions obtained using a low-cost replacement model (surrogate). Here, data-driven surrogates are utilized, which are obtained by approximating sampled EM data using co-kriging. The resulting surrogate model is fast, so the antenna evaluation cost is negligible compared to EM analysis. This is of fundamental importance, especially in the context of global optimization, which requires a large number of system simulations. EM analysis is only executed occasionally to verify the quality of newly created infill points, and to update the surrogate model, which gradually becomes a better and better representation of the antenna at hand.

### Design problem formulation

The statement of the design task largely depends on the specific optimization goals. In this study, we concentrate on optimizing the input characteristics of multi-band antennas, aiming to position the resonances at specified locations in terms of (target) frequencies ***F***_*t*_ = [*f*_*t*.1_ … *f*_*t*.*K*_]^*T*^, and improving the impedance matching therein (i.e., minimizing the reflection coefficient modulus |*S*_11_| at all frequencies *f*_*t*.*j*_, *j* = 1, …, *K*). The problem is usually framed as a minimax objective function, detailed in Table 1 alongside the required notation. Using this terminology, we can express the design task in the following form

1 $${\boldsymbol{x}}^{*} = \arg \mathop {\min }\limits_{{\boldsymbol{x}}} U({\boldsymbol{x}},{\boldsymbol{F}}_{t} )$$

**Table 1:** Multi-band antenna optimization for impedance matching improvement.

Symbol	Meaning	Comment
***x*** = [*x*_1_ … *x*_*n*_]^*T*^	Vector of antenna design parameters	Typically, the variables are antenna geometry parameters (dimensions in mm)
*S*_11_(***x***,*f*)	Antenna reflection coefficient at the design ***x*** and frequency *f*	Reflection coefficient is a complex number; in the design process we handle its modulus |*S*_11_|, expressed in decibels
*F*_*t*_ = [*f*_*t*.1_ … *f*_*t*.*K*_]^*T*^	Vector of target operating frequencies	Frequencies describing a required allocation of antenna resonances
*U*(***x***,***F***_*t*_)	Objective function to be minimized in the design process	Function quantifying the design quality, here, defined as $U({\boldsymbol{x}},{\boldsymbol{F}}_{t} ) = \mathop {\max }\limits_{{\boldsymbol{x}}} \left\{ {|S_{11} ({\boldsymbol{x}},f_{t.1} )|,...,|S_{11} ({\boldsymbol{x}},f_{t.K} )|} \right\}$

The problem (1) can be generalized to handle other types of goals, for example, impedance matching improvement over fractional bandwidths centred at *f*_*t*.*j*_, maximization of the impedance bandwidth, minimization of axial ratio, improvement of the antenna gain, etc. As mentioned earlier, the specific task considered here is a representative case, which is often dealt with in practice. Here, it is employed to demonstrate the global search framework discussed in the paper. At the same time, it should be emphasized that the considered design problem is a nominal one, i.e., no parameter deviations (e.g., fabrication tolerances or other types of uncertainties) are considered.

### Response features

The primary bottleneck in simulation-driven antenna optimization is the extensive computational expense linked to numerous EM analyses, which are essential for numerical search processes. This cost is notably substantial for local optimization and escalates significantly when employing global algorithms.

**Figure 1:**
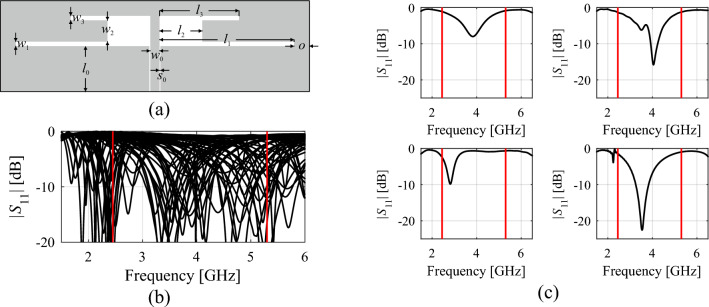
Dual-band antenna and challenges associated with its global optimization: (**a**) geometry, (**b**) family of responses corresponding to random designs allocated over the assumed design space, (**c**) selected characteristics. The target operating frequencies: 2.45 GHz and 5.3 GHz are indicated by vertical lines. For most of designs of (**b**) and (**c**), local optimization oriented towards matching improvement at the target frequencies would fail. Proper allocation of the antenna resonances requires global search.

Global search involves exploring the entire parameter space, a challenging task due to its vastness in both dimensionality and parameter ranges. The nonlinear nature of antenna outputs, especially in multi-band antennas, further complicates this task. As shown in Fig. 1 for an exemplary planar antenna, the sharp resonant input characteristics pose difficulties. If pursuing a local search approach for problem (1), it would fail if initiated from most designs depicted in Fig. 1b. Additionally, creating a dependable surrogate model that accurately represents these resonant characteristics is complicated due to the response shape.

One way to address these challenges is through a response feature approach^81^, which involves reformulating the design task using characteristic points of the antenna outputs^78^. This method leverages the weakly nonlinear dependency of the characteristic point coordinates (such as frequencies and levels) on antenna geometry parameters^77–81^. Utilizing this approach regularizes the objective function, facilitating faster convergence of the optimization process^81^. It also enables quasi-global search capabilities^83^ and reduces the amount of training points required to build a dependable metamodel^82^.

**Figure 2:**
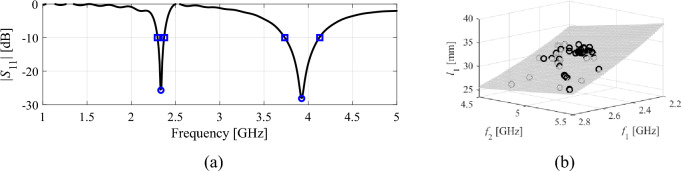
Dual-band dipole antenna: (**a**) response features corresponding to antenna resonances (o) and –10 dB reflection levels (open square); note that some of the feature points may not exist depending on the design (e.g., one of the resonances being outside the simulation frequency range); (**b**) relationship between the operating frequencies and selected geometry parameter. Note that clear trend is visible, as emphasized using a least-square regression model (gray dots). The latter demonstrates weakly-nonlinear relations between the response feature coordinates and geometry parameters.

Feature points must align with the design objectives^78^. For impedance matching improvement of multi-band devices, selecting frequency and level allocation of antenna resonances is apt. This is exemplified in Fig. 2, where additional feature points related to the –10 dB levels of |*S*_11_| are depicted, aiding in enlarging antenna bandwidth. As shown in Fig. 2(b), the coordinates of feature points display relatively simple patterns (in contrast to complete antenna characteristics; for an in-depth discussion, refer to^78,81^).

In the remaining part of this work, we use ***f***_*P*_(***x***) = [***f***_*f*_(***x***)^*T*^ ***f***_*L*_(***x***)^*T*^]^*T*^, where ***f***_*f*_(***x***) = [*f*_*f*.1_(***x***) … *f*_*f.K*_(***x***)]^*T*^ and ***f***_*L*_(***x***) = [*f*_*L*.1_(***x***) … *f*_*L.K*_(***x***)]^*T*^, to denote a response feature vector, its horizontal and vertical coordinates, respectively. The feature-oriented merit function takes the form of

2 $$U_{F} ({\boldsymbol{x}},{\boldsymbol{f}}_{P} ,{\boldsymbol{F}}_{t} ) = \mathop {\max }\limits_{{\boldsymbol{x}}} \left\{ {f_{L.1} {(}{\boldsymbol{x}}{)},...,f_{L.K} {(}{\boldsymbol{x}}{)}} \right\} + \beta ||{\boldsymbol{f}}_{f} - {\boldsymbol{F}}_{t} ||^{2}$$

in which β ||***f***_*f*_ – ***F***_*t*_||^2^ is a regularization factor, implemented to facilitate the adjustment of resonant frequencies towards their intended values. While the specific value of *β* is not overly critical, it should be selected to ensure that the regularization term significantly contributes in scenarios where resonant frequencies exhibit considerable misalignment (here, we set β = 100). It can be noted that formulation (2) is different than the minimax one of Table 1, yet the optimum solutions with respect to both are equivalent assuming that the requested operating frequencies can be reached.

### Variable-resolution EM models

**Figure 3:**
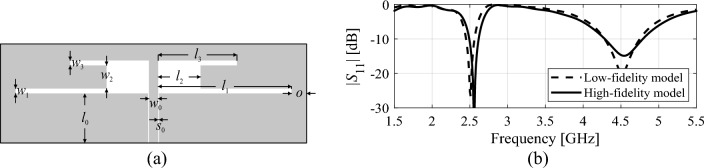
Variable-fidelity models: (**a**) an exemplary dual-band antenna, (**b**) its reflection responses evaluated using the low-fidelity EM model (- - -) and the high-fidelity one (—). In the case shown in the picture, the simulation time of the high-fidelity model is about 90 s, whereas the evaluation of the low-fidelity model only takes about 25 s.

Low-fidelity models can be used to accelerate design optimization procedures primarily by reducing the time required for system evaluation. The trade-off is in the loss of accuracy (cf. Fig. 3), which has to be compensated for through appropriate correction (e.g., space mapping^84,85^). When dealing with antenna structures, coarse-discretization EM analysis stands out as a versatile and effective low-fidelity modeling approach^86^. The actual speedup depends on the particular antenna structure, and the acceleration factors vary from less than three to over ten, assuming that the reduced-resolution model still renders essential details of antenna response.

In subsequent discussions, the low-resolution model, termed ***R***_*c*_(***x***), will have a dual role: (i) generating a set of random observables for parameter space pre-screening, and (ii) building the initial surrogate model using kriging (refer to Section "Kriging interpolation. Co-Kriging surrogates"). As the process progresses, low-resolution data will merge with the gathered high-fidelity points to create an enhanced surrogate (utilizing co-kriging, cf. Section "Kriging interpolation. Co-Kriging Surrogates"). We will employ a notation ***R***_*f*_(***x***) to refer to the high-fidelity model.

### Kriging interpolation. Co-Kriging Surrogates

Here, we briefly recall kriging and co-kriging interpolation^87^, utilized to build replacement models employed as predictors in the optimization procedure developed in this paper.

Let {***x***_*Bc*_^(*k*)^,***R***_*c*_(***x***_*Bc*_^(*k*)^)}_*k* = 1, …, *NBc*_, denote the low-resolution dataset consisting of parameter vectors ***x***_*Bc*_^(*k*)^ and the corresponding antenna responses evaluated at the low-fidelity EM simulation level. We will also denote by {***x***_*Bf*_^(*k*)^,***R***_*f*_(***x***_*Bf*_^(*k*)^)}_*k* = 1, …, *NBf*_, the high-fidelity dataset, obtained through high-fidelity EM analysis at the locations ***x***_*Bf*_^(*k*)^.

The details concerning kriging and co-kriging models ***s***_*KR*_(***x***) and ***s***_*CO*_(***x***) can be found in Table 2. Co-kriging surrogate blends together (i) a kriging model ***s***_*KRc*_ established using the low-resolution data (*X*_*Bc*_, ***R***_*c*_(*X*_*Bc*_)), (ii) ***s***_*KRf*_ determined using the residuals (*X*_*Bf*_, ***r***), where ***r*** = ***R***_*f*_(*X*_*Bf*_) – *ρ*⋅***R***_*c*_(*X*_*Bf*_); here *ρ* is included in the Maximum Likelihood Estimation (MLE) of the second model^88^. ***R***_*c*_(*X*_*Bf*_) can be taken as ***R***_*c*_(*X*_*Bf*_) ≈ ***s***_*KRc*_(*X*_*Bf*_) in case the relevant low-fidelity data is not available. Both models use the same correlation function (cf. Table 2).

**Table 2:** Kriging and co-kriging surrogate models.

Model	Component	Analytical form
Kriging	Model formulation	${\boldsymbol{s}}_{KR} ({\boldsymbol{x}}) = {\boldsymbol{M}}\gamma + r({\boldsymbol{x}}) \cdot {{\varvec{\Psi}}}^{ - 1} \cdot ({\boldsymbol{R}}_{c} (X_{Bc} ) - {\boldsymbol{F}}\gamma )$ where ***M*** is a *N*_*Bc*_ × *t* model matrix of *X*_*Bc*_, whereas ***F*** is a 1 × *t* vector of the evaluation point ***x*** (*t* is the number of terms used in the regression function^88^)
	Regression function coefficients	$\gamma = (X_{Bc}^{T} {{\varvec{\Psi}}}^{ - 1} X_{Bc} )^{ - 1} X_{Bc} {{\varvec{\Psi}}}^{ - 1} {\boldsymbol{R}}_{f} (X_{Bc} )$
	1 × *N*_*Bc*_ vector of correlations between ***x*** and *X*_*Bc*_	$r({\boldsymbol{x}}) = (\psi ({\boldsymbol{x}},{\boldsymbol{x}}_{Bc}^{(1)} ),...,\psi ({\boldsymbol{x}},{\boldsymbol{x}}_{Bc}^{{(N_{Bc} )}} ))$
	Correlation matrix	***Ψ*** = [Ψ_*i*,*j*_] is a correlation matrix, where Ψ_*i*,*j*_ = *ψ*(***x***_*Bf*_^(*i*)^,***x***_*Bf*_^(*j*)^)
	Correlation function	$\psi ({\boldsymbol{x}},{\boldsymbol{x}}{\prime} ) = \exp \left( {\sum\nolimits_{k = 1}^{n} { - \theta_{k} |x^{k} - x^{^{\prime}k} |^{P} } } \right)$
	Model identification: finding hyperparameters *θ*_*k*_, *k* = 1, …, *n*, using Maximum Likelihood Estimation (MLE^88^)	$(\theta_{1} ,...,\theta_{n} ) = \arg \mathop {\min }\limits_{{\theta_{1} ,...,\theta_{n} }} \left[ { - (N_{Bf} /2)\ln (\hat{\sigma }^{2} ) - 0.5\ln (|\Psi |)} \right]$ where $\hat{\sigma }^{2} = ({\boldsymbol{R}}_{f} (X_{Bf} ) - F\alpha )^{T} {{\varvec{\Psi}}}^{ - 1} ({\boldsymbol{R}}_{f} (X_{Bf} ) - F\alpha )/N_{Bf}$ and |***Ψ***| is the determinant of ***Ψ***. In practice, a Gaussian correlation function (*P* = 2) is often employed, as well as ***F*** = [1 … 1]^*T*^ and ***M*** = 1
Co-kriging	Model formulation	${\boldsymbol{s}}_{CO} ({\boldsymbol{x}}) = {\boldsymbol{M}}\gamma + r({\boldsymbol{x}}) \cdot {{\varvec{\Psi}}}^{ - 1} \cdot ({\boldsymbol{r}} - {\boldsymbol{F}}\gamma )$
	Vector of correlations	$r({\boldsymbol{x}}) = [\rho \cdot \sigma_{c}^{2} \cdot r_{c} ({\boldsymbol{x}}),\rho^{2} \cdot \sigma_{c}^{2} \cdot r_{c} ({\boldsymbol{x}},X_{{B_{f} }} ) + \sigma_{d}^{2} \cdot r_{d} ({\boldsymbol{x}})]$
	Correlation matrix	$${{\varvec{\Psi}}} = \left[ {\begin{array}{*{20}c} {\sigma_{c}^{2} {{\varvec{\Psi}}}_{c} (X_{Bc} ,X_{Bc} )} & {\rho \,\sigma_{c}^{2} {{\varvec{\Psi}}}_{c} (X_{Bc} ,X_{Bf} )\,} \\ {\rho \,\sigma_{c}^{2} {{\varvec{\Psi}}}_{c} (X_{Bf} ,X_{Bc} )} & {\rho^{2} \sigma_{c}^{2} {{\varvec{\Psi}}}_{c} (X_{Bf} ,X_{Bf} ) + \sigma_{d}^{2} {{\varvec{\Psi}}}_{d} } \\ \end{array} } \right]$$ where and ***M*** = [*ρ****M***_*c*_ ***M***_*d*_] where (***F***_*c*_, *σ*_*c*_, ***Ψ***_*c*_, ***M***_*c*_) and (***F***_*d*_, *σ*_*d*_, ***Ψ***_*d*_, ***M***_*d*_) are matrices obtained from ***s***_*KRc*_ and ***s***_*KRf*_, respectively^88^; parameter *ρ* is included in the MLE during model identification

### Parameter space pre-screening. construction of the initial surrogate

In the proposed methodology outlined in this study, the search initiates by creating an ensemble set of random parameter vectors evaluated with a low-resolution EM model. Eligible samples, capable of extracting response features, are employed to build the initial surrogate model. This approach operates within the response feature space, enabling a regularization of the cost function, which significantly diminishes the amount of data points required to set up a dependable metamodel.

The metamodel ***s***^(0)^(***x***) is built to make predictions about coordinates of the feature points, i.e., their frequencies and levels. We have

3 $${\boldsymbol{s}}^{(0)} ({\boldsymbol{x}}) = \left[ {\left[ {s_{f.1}^{(0)} ({\boldsymbol{x}})\;...\;s_{f.K}^{(0)} ({\boldsymbol{x}})} \right]^{T} \;\left[ {s_{L.1}^{(0)} ({\boldsymbol{x}})\;...\;s_{L.K}^{(0)} ({\boldsymbol{x}})} \right]^{T} } \right]^{T}$$

**Figure 4:**
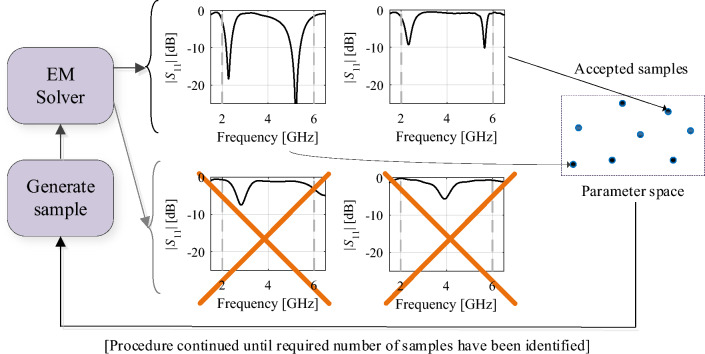
Generation of random observables for initial surrogate model construction. Only samples for which the corresponding antenna responses have resonances within the target frequency range are selected.

**Figure 5:**
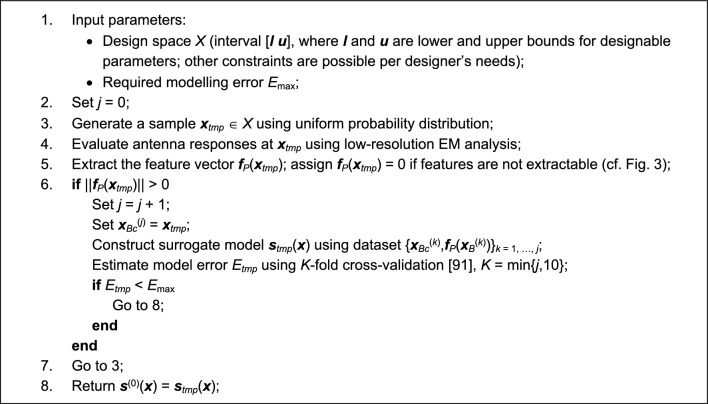
Generating the training data set for initial surrogate model construction.

The model ***s***^(0)^ is identified using kriging interpolation^89^ (cf. Section "Kriging interpolation. Co-Kriging surrogates"). The training dataset consist of the vectors ***x***_*Bc*_^(*j*)^, *j* = 1, …, *N*_*init*_, and the corresponding feature vectors ***f***_*P*_(***x***_*Bc*_^(*j*)^), extracted from low-fidelity EM simulation data. The data points are generated sequentially, and only the points with extractable characteristic points (cf. Fig. 4) are included. The number *N*_*init*_ is set to guarantee sufficient reliability of the metamodel. The acceptance threshold *E*_max_ for a relative RMS error^73^ a user-defined parameter. Figure 5 summarizes the procedure for generating the training data points. In practice, *N*_*init*_ is between 50 and 200, depending on the problem complexity. The actual number of random trials (i.e., low-fidelity EM model simulations) required to produce *N*_*init*_ acceptable samples is two to three times larger than *N*_*init*_.

### Generating infill points using PSO. Co-Kriging surrogate

As discussed in Section "Kriging interpolation. Co-Kriging surrogates", the initial metamodel ***s***^(0)^ is identified using *N*_*init*_ low-resolution data samples obtained through parameter space pre-screening. In the core stage of the search procedure, the metamodel is subject to refinement with the use of high-fidelity samples ***x***_*f*_^(*i*)^, *i* = 1, 2, …, obtained by solving

4 $${\boldsymbol{x}}_{f}^{(i + 1)} = \arg \mathop {\min }\limits_{{{\boldsymbol{x}} \in X}} U_{F} ({\boldsymbol{x}},{\mathbf{s}}^{(i)} ({\boldsymbol{x}}),{\boldsymbol{F}}_{t} )$$

where ***s***^(*j*)^, *j* = 1, 2, …, are co-kriging surrogates (cf. Section "Parameter space pre-screening. construction of the initial surrogate") constructed using low-resolution dataset {***x***_*Bc*_^(*j*)^,*** f***_*P*_(***x***_*Bc*_^(*j*)^)}, *j* = 1, …, *N*_*init*_, as well as high-fidelity dataset consisting of the high-fidelity samples acquired until the *i*th iteration, {***x***_*f*_^(*j*)^,*** f***_*P*_(***x***_*f*_^(*j*)^)}, *j* = 1, …, *i*.

The current metamodel ***s***^(*i*)^ acts as a predictor, optimized to yield the subsequent iteration points. The solution to (4) is found in a global sense using a particle swarm optimizer (PSO)^91^. Given a fast metamodel, the selection of the search routine is of minor significance, and the optimization process can be executed using relatively large computational budget. Also, formulating the problem using response features facilitates the task even further. The regularization term within the objective function (2) essentially promotes a nearly monotonic behaviour concerning the gap between the current and requested center frequencies of the antenna being designed. In machine learning terms, the generation of infill points as described in (4) is akin to using predicted improvements in the objective function as the infill criterion ^92^.

It should be reiterated that as the proposed optimization framework is intended to be a global search engine, it is essential that the infill points are generated globally. As a matter of fact, for this purpose, PSO might be replaced by any bio-inspired algorithm because the global optimization stage is carried out at the level of the fast surrogate model. Consequently, most metaheuristic algorithms would perform similarly as there is no practical limit on the computational budget when solving the sub-problem (4).

The search process is terminated if the distance between subsequent iteration points is sufficiently reduced (i.e., ||***x***^(*i*+1)^ – ***x***^(*i*)^||< *ε*), or there was no improvement of the cost function over the last *N*_*no_improve*_ iterations (whichever occurs first). Both conditions essentially control the optimization process resolution. In our verification experiments (cf. Section "Verification and benchmarking"), the termination parameters are set as *ε* = 10^–2^ and *N*_*no_improve*_ = 10. It should be noted that 10^–2^ corresponds to a precise optimum allocation as for antenna dimensions expressed in millimeters, 0.01 is a small number, which is at the level of fabrication capabilities for standard manufacturing procedures.

### Optimization framework

The global optimization framework suggested in this study is summarized in this section. Table 3 gathers the control parameters, which are only three. Two of these are related to the termination condition and have already been discussed in Section "Generating infill points using PSO. Co-Kriging surrogate". The parameter *E*_max_ is an acceptance threshold concerning the relative RMS error of the initial surrogate. Here, it is set to two percent; however, this value is not critical. A value below ten percent suffices, due to the fact that the surrogate is rendered using response features, resulting in a relatively regular functional landscape, unlike the more intricate landscape of complete antenna characteristics. Hence, the algorithm setup is quite straightforward. To emphasize this aspect, identical values of control parameters are used for all demonstration case studies investigated in Section "Verification and benchmarking".

**Table 3:** Control parameters of the proposed global variable-resolution optimization framework.

Parameter	Meaning	Default value
*E*_max_	Maximum value of relative RMS error of the initial surrogate model (error estimated using cross-validation), cf. Section "Parameter space pre-screening. construction of the initial surrogate"	2%
*ε*	Termination threshold for convergence in argument, cf. Section "Generating infill points using PSO. Co-Kriging surrogate"	10^–2^
*N*_*no_improve*_	Termination threshold for no objective function value improvement, cf. Section "Generating infill points using PSO. Co-Kriging surrogate"	10

**Figure 6:**
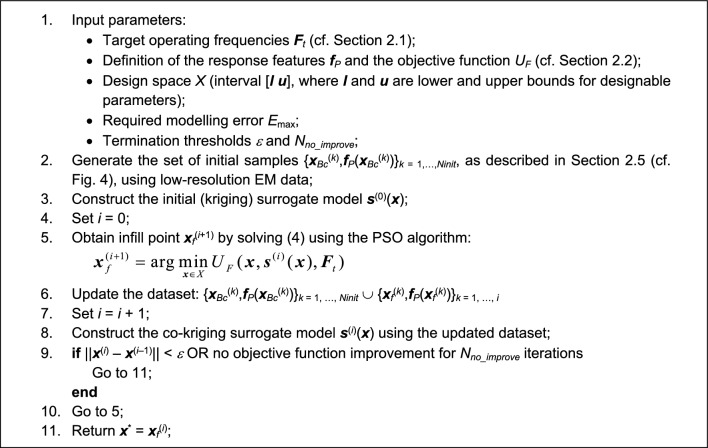
Pseudocode of the proposed global optimization algorithm for multi-band antennas.

**Figure 7:**
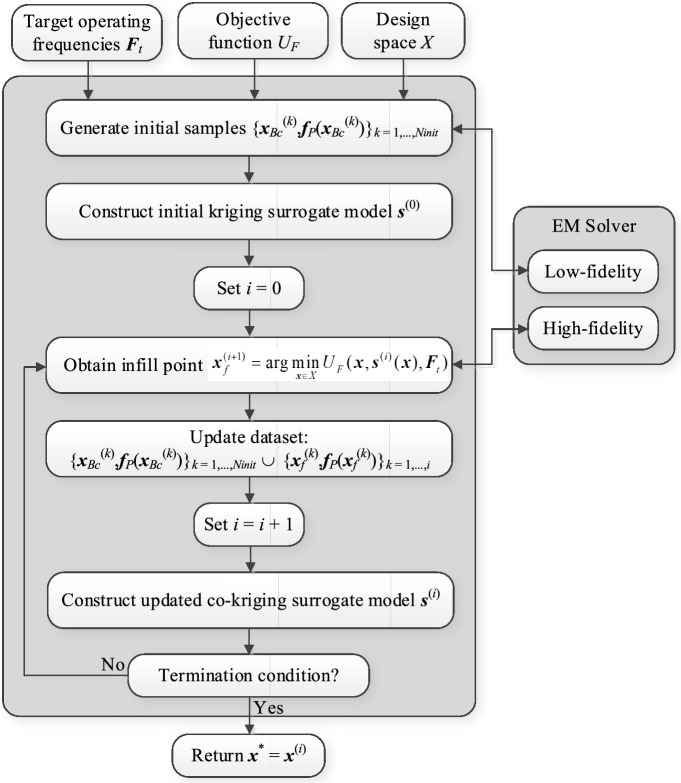
Flow diagram of the proposed global optimization algorithm for multi-band antennas.

A summary of the operating steps of the procedure can be found in Fig. 6. Using the input data, as specified in Step 1, the pre-screening stage is executed as discussed in Section "Parameter space pre-screening. construction of the initial surrogate" (Step 2). The initial surrogate model constructed in Step 3 is used as a predictor for generating the first high-fidelity infill point (Step 5). The metamodel is updated (Steps 6 and 8), and the entire infill process is continued until convergence. As a supplementary explanation, Fig. 7 provides the flow diagram of the presented algorithm.

The complete algorithm including all its components (pre-screening, CST-Matlab socket, PSO algorithm, etc.) has been implemented in Matlab^98^. The kriging and co-kriging models were implemented using Matlab-based SUMO toolbox developed at Ghent University, Belgium^99^.

## Verification and benchmarking

The effectiveness of the algorithm detailed in Section "Verification and benchmarking" is validated through three distinct multi-band antennas. We aim at gauging how the integration of variable-resolution models and response features influences the algorithm's global search capabilities and computational efficiency. Our benchmarks encompass multiple-start local search, direct EM-driven nature-inspired optimization (using PSO), and two machine learning algorithms: one operating directly with complete antenna responses, the other being a feature-based framework involving the high-resolution computational model. This comparative approach enables us to validate the multimodal nature of the design tasks and verify the impact and relevance of the mechanisms employed (response features and variable-fidelity simulations). The subsequent section organization is as follows: the verification cases are highlighted in Section "Verification antennas", Section "Setup and results" discusses the setup of the numerical experiments along with the results, and finally, Section "Discussion" offers an analysis of the results and the algorithm's performance.

It should be emphasized that experimental validation of the antenna structures produced by the proposed and the benchmark algorithms is out of the scope of this study and will not be considered. The reason is that the ultimate antenna representation is the computational model implemented and evaluated using a full-wave EM solver (here, CST Microwave Studio). Experimental validation of antennas generated using any optimization procedure would be equivalent to validating the accuracy of the simulation software, rather than the optimization procedures. Interested reader may find experimental results in the source works (e.g.,^80,93,94^).

### Verification antennas

The proposed algorithm's validation centers around the following three microstrip antenna structures:

- A dual-band uniplanar coplanar-waveguide (CPW)-fed dipole antenna^93^ (Antenna I);
- A triple-band uniplanar CPW-fed dipole^80^ (Antenna II);
- A triple-band patch antenna with a defected ground^94^ (Antenna III).

**Figure 8:**
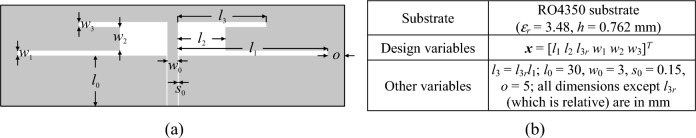
Antenna I^93^: (**a**) geometry, (**b**) essential parameters.

**Figure 9:**
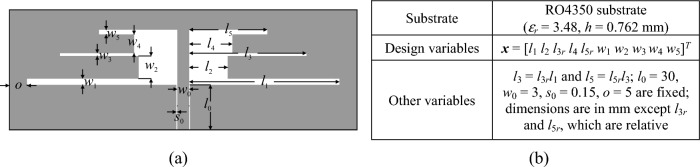
Antenna II^80^: (**a**) geometry, (**b**) essential parameters.

**Figure 10:**
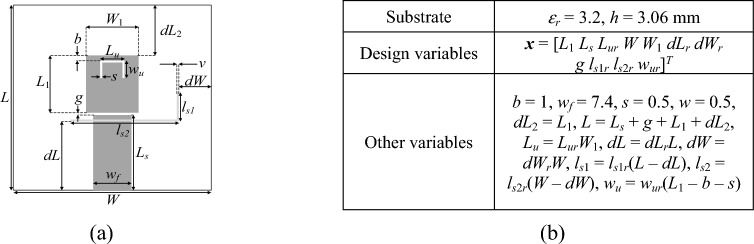
Antenna III^94^: (**a**) geometry, the light-shade grey denotes a ground-plane slot, (**b**) essential parameters.

The antenna geometries have been shown in Figs. 8, 9, 10, which also provide information about the essential parameters (design variables, material parameters of the substrate). All EM simulation models are implemented and assessed using CST Microwave Studio^95^, using the time-domain solver. All computations have been performed using the following hardware setup: micro-server machine with two 2.2 GHz Intel Xeon processors with 20 computing cores (40 logical processors), and 64 GB RAM. To acquire the low-resolution models, the structures' discretization density is decreased compared to their original high-fidelity representations. Specifics on the typical mesh density and simulation times are available in Table 4.

**Table 4:** Computational models for Antennas I through III.

Antenna	EM simulation model^#^			
	Low-fidelity ***R***_*c*_		High-fidelity ***R***_*f*_	
	Discretization density (# of mesh cells)	Simulation time [s]	Discretization density (# of mesh cells)	Simulation time [s]
I	~ 60,000	25	~ 410,000	92
II	~ 71,000	35	~ 270,000	80
III	~ 160,000	42	~ 800,000	165

^#^Reported simulation times refer to overall model evaluation times, which include updating antenna parameters withing the computational model, re-building the structure, adaptive meshing, as well as actual time-domain simulation.

The objective is to align the antenna resonant frequencies with specified targets and enhance impedance matching at those frequencies, as described in Section "Design problem formulation". For more details, refer to Table 5, which offers insights into the parameter spaces. These spaces are notably extensive, particularly regarding parameter ranges. On average, the upper-to-lower bound ratio stands at 4.2, 8.4, and 2.6 for Antennas I, II, and III, respectively.

**Table 5:** Target operating frequencies and parameter spaces for Antennas I through III.

Antenna	Target operating frequencies [GHz]	Parameter space *X* (lower bounds ***l*** and upper bounds ***u***)
I	***f***_*t*_ = [2.45 5.3]^*T*^	***l*** = [15 3 0.35 0.2 1.8 0.5]^*T*^ ***u*** = [50 12 0.85 1.5 4.3 2.7]^*T*^
II	***f***_*t*_ = [2.45 3.6 5.3]^*T*^	***l*** = [20 3 0.6 3 0.6 0.2 0.2 0.2 0.2 0.2]^*T*^ ***u*** = [50 5 0.85 5 0.85 2.2 4.2 2.2 4.2 2.2]^*T*^
III	***f***_*t*_ = [3.5 5.8 7.5]^*T*^	***l*** = [10 17 0.2 45 5 0.4 0.15 0.2 0.1 0.5 0.1]^*T*^ ***u*** = [16 25 0.6 55 15 0.5 0.3 0.8 0.4 0.65 0.5]^*T*^

### Setup and results

To enable verification, Antennas I through III were optimized by means of the proposed algorithm, set up as indicated in Table 3 (*E*_max_ = 2%, *ε* = 10^–2^, and *N*_*no_improve*_ = 10). Table 6 summarizes the benchmark algorithms utilized in the comparative experiments. These include PSO (as a popular metaheuristic procedure), multiple-start gradient search (utilized to underscore the necessity of global search for the outlined test cases), as well as two machine learning algorithms. The first one is a surrogate-assisted kriging-based procedure working with the complete antenna responses, and utilizing predicted objective function improvement as the infill criterion. The second is essentially the feature-based algorithm of Section "Global antenna optimization by response features and variable-resolution simulation models" but using a single EM simulation model (here, ***R***_*f*_(***x***)) at all design optimization stages. These algorithms are used to demonstrate the relevance of incorporating response features and variable-resolution models. The control parameter *E*_max_ was set to 10% for Algorithm III because the construction of a reliable metamodel for antenna frequency characteristics is considerably more challenging than representing feature point coordinates. Also, in this case, the maximum computational budget of 400 initial samples was assumed (in case it is not possible to reach the *E*_max_ threshold).

**Table 6:** Benchmark algorithms.

Algorithm	Algorithm type	Setup
I	Particle swarm optimizer (PSO)	Swarm size *N* = 10, standard control parameters (*χ* = 0.73, *c*_1_ = *c*_2_ = 2.05); number of iterations set to 50 (version I) and 100 (version II)
II	Trust-region gradient based optimizer^96^	Random initial design, response gradients estimated using finite differentiation, termination criteria based on convergence in argument and reduction of the trust region size^96^
III	Machine learning algorithm operating on complete antenna characteristics	Algorithm highlights: Initial surrogate set up to ensure relative RMS error not higher than 10% with the maximum number of training samples equal to 400; Optimization based on processing the antenna frequency characteristics (unlike response features in the proposed procedure); Infill criterion: minimization of the predicted objective function^92^
IV	Feature-based machine learning algorithm utilizing high-fidelity EM simulations only	Algorithm highlights: Surrogate model constructed at the level of response features; Optimization process only uses high-fidelity EM simulations; Infill criterion: minimization of the predicted objective function^92^

Each algorithm is executed ten times, and the results statistics are reported. This is necessary due to the stochastic nature of the search process. The numerical findings are summarized in Tables 7, 8, and 9. The considered performance figures include the design quality measured using the average objective function value, the computational cost expressed in terms of the number of equivalent high-fidelity EM simulations, and the success rate, which is the number of algorithm runs (out of ten) for which the algorithm allocated the antenna resonances at the intended targets.

**Table 7:** Optimization results for Antenna I.

Optimization algorithm		Performance figure			
		Average objective function value [dB]	Computational cost^$^	Relative speedup^&^	Success rate^#^
Algorithm I: PSO	50 iterations	− 18.2	500 [12.8 h]	87%	9/10
	100 iterations	− 19.3	1000 [25.6 h]	93%	10/10
Algorithm II: Trust-region gradient-based algorithm		− 13.5	84.2 [2.2 h]	22%	6/10
Algorithm III: machine learning algorithm processing complete antenna responses		− 20.7	457.8 [11.7 h]	86%	10/10
Algorithm IV: feature-based machine learning algorithm using high-fidelity EM model		− 20.3	92.3 [2.4 h]	29%	10/10
Proposed algorithm		− 23.9	65.3 [1.7 h]	–	10/10

^$^The cost expressed in terms of the equivalent number of high-fidelity EM simulations of the antenna structure under design. Values in brackets is the total CPU time in hours.

^#^Number of algorithms runs at which the operating frequencies were allocated in the vicinity of the target frequencies.

^&^Relative speedup obtained by the proposed method with respect to the benchmark techniques.

**Table 8:** Optimization results for Antenna II.

Optimization algorithm		Performance figure			
		Average objective function value [dB]	Computational cost^$^	Relative speedup^&^	Success rate^#^
Algorithm I: PSO	50 iterations	− 10.8	500 [11.1 h]	76%	5/10
	100 iterations	− 13.8	1,000 [22.2 h]	88%	8/10
Algorithm II: Trust-region gradient-based algorithm		− 7.8	105.8 [2.4 h]	− 14%	4/10
Algorithm III: machine learning algorithm processing complete antenna responses		− 13.5	470.0 [10.4 h]	74%	10/10
Algorithm IV: feature-based machine learning algorithm using high-fidelity EM model		− 13.7	251.9 [5.6 h]	52%	10/10
Proposed algorithm		− 13.5	121.2 [2.7 h]	–	10/10

^$^The cost expressed in terms of the equivalent number of high-fidelity EM simulations of the antenna structure under design. Values in brackets is the total CPU time in hours.

^#^Number of algorithms runs at which the operating frequencies were allocated in the vicinity of the target frequencies.

^&^Relative speedup obtained by the proposed method with respect to the benchmark techniques.

**Table 9:** Optimization results for Antenna III.

Optimization algorithm		Performance figure			
		Average objective function value [dB]	Computational cost^$^	Relative speedup^&^	Success rate^#^
Algorithm I: PSO	50 iterations	− 12.3	500 [22.9 h]	82%	6/10
	100 iterations	− 14.2	1,000 [45.8 h]	91%	8/10
Algorithm II: Trust-region gradient-based algorithm		− 12.1	125.4 [5.7 h]	28%	4/10
Algorithm III: machine learning algorithm processing complete antenna responses		− 14.2	473.0 [21.7 h]	81%	7/10
Algorithm IV: feature-based machine learning algorithm using high-fidelity EM model		− 17.9	347.0 [15.9 h]	74%	10/10
Proposed algorithm		− 15.3	89.8 [4.1 h]	–	10/10

^$^The cost expressed in terms of the equivalent number of high-fidelity EM simulations of the antenna structure under design. Values in brackets is the total CPU time in hours.

^#^Number of algorithms runs at which the operating frequencies were allocated in the vicinity of the target frequencies.

^&^Relative speedup obtained by the proposed method with respect to the benchmark techniques.

**Figure 11:**
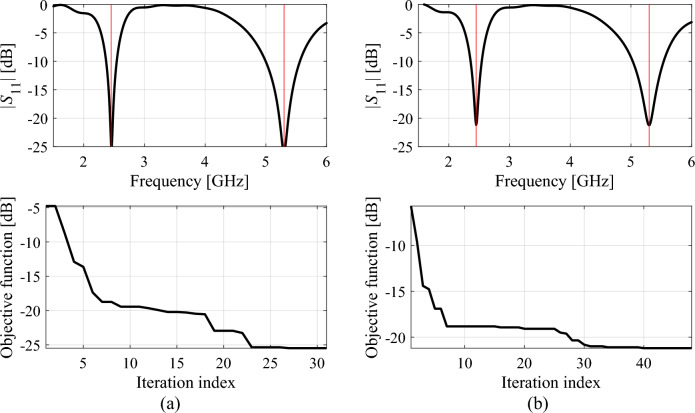
Reflection responses of Antenna I at the designs obtained using the proposed global optimization algorithm (top) and evolution of the objective function value (bottom), shown for selected algorithm runs: (**a**) run 1, (**b**) run 2. The iteration counter starts after constructing the initial surrogate model. Vertical lines mark the target operating frequencies, here 2.45 GHz and 5.3 GHz.

**Figure 12:**
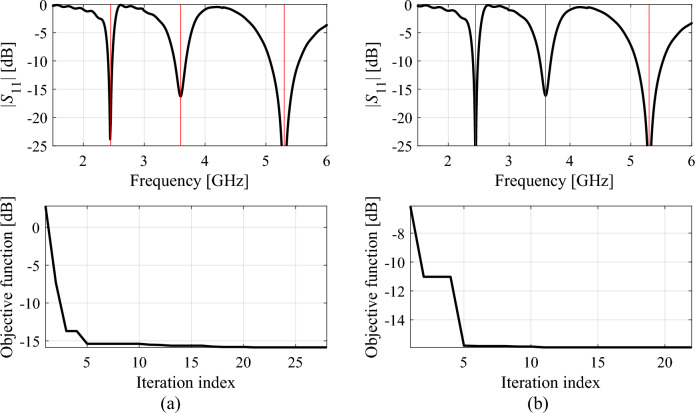
Reflection responses of Antenna II at the designs obtained using the proposed global optimization algorithm (top) and evolution of the objective function value (bottom), shown for selected algorithm runs: (**a**) run 1, (**b**) run 2. The iteration counter starts after constructing the initial surrogate model. Vertical lines mark the target operating frequencies, here 2.45 GHz, 3.6 GHz, and 5.3 GHz.

**Figure 13:**
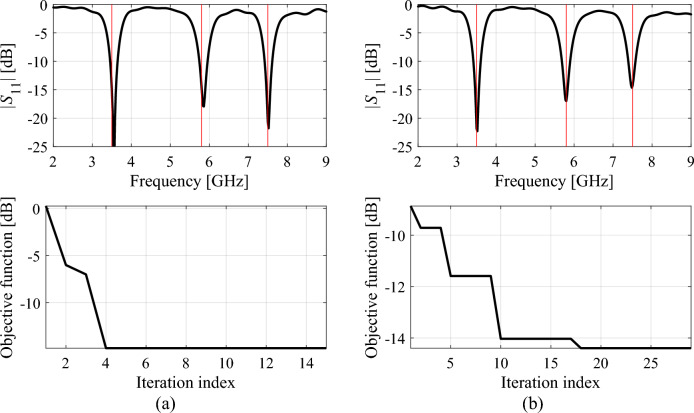
Reflection responses of Antenna III at the designs obtained using the proposed global optimization algorithm (top) and evolution of the objective function value (bottom), shown for selected algorithm runs: (**a**) run 1, (**b**) run 2. The iteration counter starts after constructing the initial surrogate model. Vertical lines mark the target operating frequencies, here 3.5 GHz, 5.8 GHz, and 7.5 GHz.

**Figure 14:**
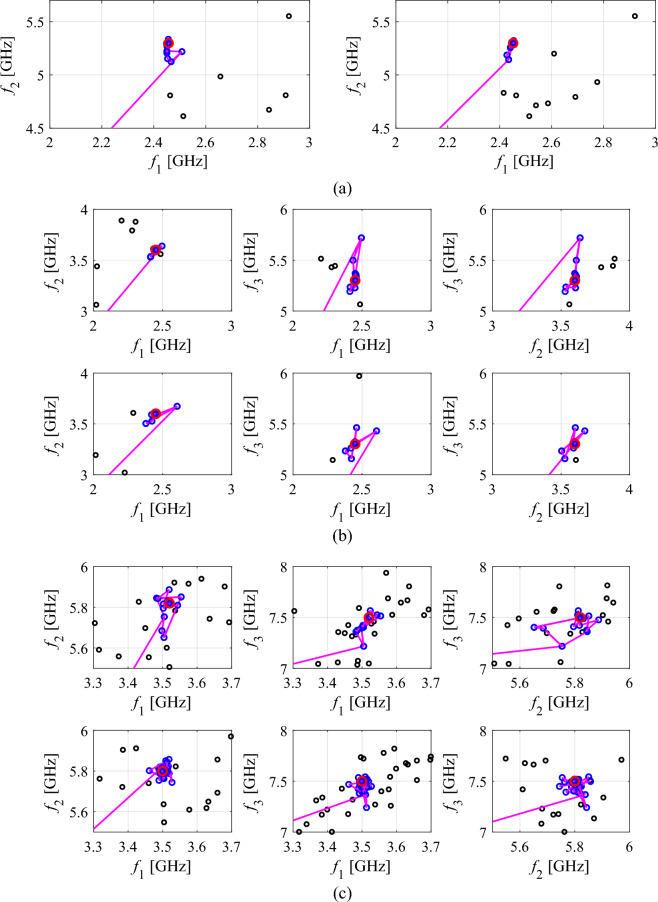
Optimization history in the feature space for: (**a**) Antenna I, (**b**) Antenna II, (**c**) Antenna III. Shown are the plots corresponding to the two selected runs of the proposed algorithm as presented in Figs. 11, 12, 13 for Antennas I through III, respectively. The black and the blue dots represent the initial (low-fidelity) samples, and the infill (high-fidelity) samples, whereas the line segments illustrate the optimization path. The final solution is marked as a large circle. Figures (**b**) and (**c**) are two-dimensional projections of the three-dimensional feature space. The range of operating frequencies was adjusted to provide the details of the optimization history in the vicinity of the final solution.

Additionally, Figs. 11, 12, 13 showcase the reflection characteristics of Antennas I through III. These figures highlight the designs generated by the proposed framework during specific algorithm runs and demonstrate the evolution of the merit function over the iteration index. Moreover, Fig. 14 depicts the optimization trajectory in the feature space of antenna operating frequencies, emphasizing the same selected runs as those illustrated in Figs. 11, 12, 13. It is worth noting that the specific choice of the infill criterion (minimization of the predicted objective function) results in an increased density of infill points around the optimal design.

The analysis of the optimization history in the feature space for all considered antennas allows us to conclude that the majority of the improvement in allocation of the antenna operating frequencies occurs during the first few iterations. Afterwards, only small changes in the values of the said frequencies are observed, meaning that these are fine-tuned in the final part of the procedure. This is corroborated by the results obtained for each verification antenna structure shown in the bottom panels of Figs. 11, 12, 13, where the objective function value rapidly decreases across a few initial iterations. Whereas the reduction in the objective function value throughout the final steps of the optimization procedure is considerably smaller.

In our work, we utilize a standard setup of PSO algorithm parameters, although numerous strategies for selecting values of the control parameters of PSO algorithms have been analyzed^100,101^. We use a common approach where *χ* is equal to 0.7298 (see Table 6). Moreover, for this value of *ϕ* it is recommended that the parameters *c*_1_ and *c*_2_ sum to 4.1. Thus we use *c*_1_ = *c*_2_ = 0.5. Nevertheless, in the presented work, fine-tuning the values of the parameters is unnecessary, as we employ a PSO optimizer to optimize a fast surrogate model. Therefore, the optimization process may be carried out as long as required to ensure sufficient solution accuracy.

### Discussion

The assessment of the proposed optimization framework is consolidated based on the outcomes detailed in Section "Setup and results". Furthermore, a comparative analysis against benchmark techniques (Algorithms I through IV from Table 6) is conducted to evaluate factors such as the optimization process's reliability, design quality, and computational efficiency. Subsequent discussions delve into these crucial aspects.

Design reliability: The measure of reliability is the success rate (shown in the last column of Tables 7, 8, 9), i.e., the count of successful runs (out of ten) where the algorithm allocated the antenna resonances at the intended targets. The proposed algorithm exhibits perfect success rate in this sense, similarly as the other two machine-learning-based procedures (Algorithms III and IV). Other methods are considerably worse, which, in the case of Algorithm II is an indication of multimodality of the considered design tasks. For PSO, the results (especially the performance improvement from 50 to 100 iteration version) demonstrate that nature-inspired optimization requires considerably higher computational budget. Also, the performance of Algorithm III is inferior for Antenna III, which is the most complex task, and building a dependable replacement model of the complete antenna characteristics is much more challenging here.

Design quality: The design quality is assessed by calculating the mean value of the cost function at the final design. This value is comparable for all algorithms; the differences at the level of one to three decibels have a minor importance due to the response shape. Clearly, seemingly worse values shown by Algorithms I and II are related to the fact the average performance is displayed, which is reduced by unsuccessful runs.

Computational efficiency: The numbers in Tables 7, 8 and 9 demonstrate that the expenses entailed by the optimization process are by a great amount the lowest for our procedure, as compared to other global search algorithms. The average cost, given as the equivalent number of high-resolution EM simulations is only 65, 121, and 90, for Antenna I, II, and III, respectively. The savings with respect to Algorithm IV, which differs from the proposed one in terms of only using high-fidelity models, are as high as 30, 52, and 74 percent. This accomplishment is attributed to the integration of variable-resolution EM simulations. Also, it should be noted that the computational benefits increase with the problem complexity. The majority of antenna simulations occur in the first stage of the search procedure, specifically during the parameter space pre-screening and initial surrogate model construction, utilizing the low-fidelity model. When compared to Algorithm II (gradient search), it is noteworthy that the cost of the proposed approach aligns quite closely with local optimization, marking a significant achievement. Overall, our technique outperforms methods available in the literature in the realm of computer-aided design of antenna systems in terms of computational efficiency. Clearly, expediting optimization processes is advantageous from the point of view od speeding up the design cycles and pushing forward the state-of-the-art in antenna design automation as well as other areas where utilization of CPU-intensive simulation models is ubiquitous.

A separate note should be made about response features. While the proposed algorithm only required 77, 230, and 259 random observables in the first stage of the optimization process (and, in all cases, the assumed modeling error *E*_max_ was achieved), Algorithm III working with the complete antenna responses was unable to reach the required accuracy limit, thus, the surrogate was eventually established using 400 data samples (the allowed computational budget). Leveraging response features results in a remarkable reduction in the computational expenses during this stage of the process, presenting another advantageous facet of the presented approach.

The performance assessment formulated in the last few paragraphs demonstrates that the proposed algorithm might be a viable alternative for the existing global search techniques, at least in the realm of multi-band antenna design. Similar performance may be expected for other types of problems, assuming they can be reformulated using response features. The three most important advantages of our methodology are design reliability, computational efficiency, and a straightforward setup: the algorithm comprises only three control parameters, with two specifically relevant to the termination criteria.

## Conclusion

This article presented an algorithmic framework designed for cost-effective global optimization of multi-band antennas. Operating within a machine learning paradigm, this method operates at the level of antenna’s characteristic points and utilizes variable-resolution electromagnetic simulations. Using response features allows regularization of the objective function landscape, reducing the data needed for surrogate model construction. Variable-resolution simulations cut down computational expenses during parameter space exploration, primarily utilized in the initial stages. The simplification achieved through response features facilitates an infill strategy focused on parameter space exploitation (minimization of the predicted objective function). Extensive numerical validation across three microstrip antennas consistently demonstrates the method's competitive effectiveness, yielding satisfactory results in every algorithm run out of multiple executions performed. Benchmarking against leading methods underscores the relevance and benefits of the incorporated algorithmic techniques. The average computational expense of the search process equates to approximately ninety high-fidelity evaluations of the antenna at hand. This technique shows promise as a computationally efficient alternative to existing global search algorithms. Future work aims to broaden its scope to handle other antenna responses such as axial ratio, gain, or bandwidth. Furthermore, scalability of the proposed technique for higher-dimensional problems will be considered. Finally, possibilities to apply our method in different engineering fields (e.g., aerospace) will be considered, which is however contingent upon appropriate definition of field- and problem-dependent response features (the latter being an inherent part of design problem formulation).

## Data Availability

The datasets generated during and/or analysed during the current study are available from the corresponding author on reasonable request.
